# TML‐6 targeting at regulation of autophagolysosomal machinery in neurons and microglia represents a novel drug for AD therapy

**DOI:** 10.1002/alz70855_096731

**Published:** 2025-12-23

**Authors:** Ih‐Jen Su, Chien Hong Lin, Chia‐Yu Hsu, Hui‐Chen Wang, Feng‐Shiun Shie

**Affiliations:** ^1^ Southern Taiwan University of Science and Technology, Tainan City, Taiwan, Taiwan; ^2^ Merry Life Biomedical Company, Ltd., Tainan City, Taiwan, Taiwan; ^3^ National Health Research Institutes, Zhunan Town, Taiwan

## Abstract

**Background:**

Alzheimer's disease (AD) features a characteristic accumulation of amyloid beta (Aβ) in neurons and extracellular senile plaques in hippocampus. Although many mechanisms of AD have been proposed, the recent theory of faulty of **autophagolysosomal deacidification in neuron** as a pivotal AD mechanism received wide recognition. TML‐6, a synthetic curcumin analog has been identified to be a potential AD drug to remove Aβ, inhibit inflammation, and activate autophagic function. In this study, we further extended the studies from neurons to microglia which plays the major phagocytic and lysosomal function to remove extracellular senile plaques in AD.

**Method:**

The lysosomal integrity in microglia was damaged by the lysosomotropic reagent l‐leucyl‐l‐leucine methyl ester (LLOMe), and Aβ was used to evaluate the phagocytic lysosomal responses in primary microglia cultures.

**Result:**

TML‐6 could promote the integrity of lysosomal membrane and rescue the LLOMe‐induced lysosomal permeabilization in microglia. Data also showed that TML‐6 could enhance lysosomal acidity. A pulse‐chase study revealed enhanced phagocytic Aβ uptake and Aβ clearance by lysosomes under TML‐6 treatment.

**Conclusion:**

The activation of autophagolysosomal functions in neurons and microglias by TML‐6 to reduce intraneuronal and extracellular Aβ provides the revolutional concept to develop AD drugs. TML‐6 will represent a novel drug with total solution for AD therapy.

**Key Words**:

TML‐6; autophagolysosomal regulation; microglia; β‐amyloid clearance; Alzheimer's disease.

